# Laparoscopic restorative proctocolectomy with transanal total mesorectal excision for familial adenomatous polyposis in a 13-year-old: Case report and review of minimally invasive approaches

**DOI:** 10.1016/j.ijscr.2025.111697

**Published:** 2025-07-20

**Authors:** Hariruk Yodying

**Affiliations:** Department of Surgery, HRH Princess MahaChakri Sirindhorn Medical Center, Faculty of Medicine, Srinakharinwirot University, Nakhon Nayok, Thailand

**Keywords:** Familial adenomatous polyposis, Transanal total mesorectal excision, TaTME, Ileal pouch-anal anastomosis, Adolescent colorectal surgery, Pediatric proctocolectomy

## Abstract

**Background:**

Familial adenomatous polyposis (FAP) necessitates prophylactic colorectal surgery to prevent inevitable malignant transformation. While transanal approaches have gained acceptance in adult colorectal surgery, their application in pediatric populations remains limited. We present a young case and review current minimally invasive approaches for adolescent FAP management.

**Case presentation:**

A 13-year-old girl with genetically confirmed FAP (APC mutation c.3927_3931delAAAGA) presented with chronic lower gastrointestinal bleeding and iron deficiency anemia. Colonoscopy revealed extensive polyposis with high-grade dysplasia in multiple polyps involving both rectum and sigmoid colon. We performed laparoscopic restorative proctocolectomy with ileal pouch-anal anastomosis using transanal total mesorectal excision (TaTME) technique for rectal dissection. Total operative time was 280 min with minimal blood loss. The patient recovered uneventfully and was discharged on postoperative day 7. Diverting ileostomy was reversed at 10 weeks following contrast enema confirmation of intact anastomosis.

**Discussion:**

To our knowledge, this represents the youngest reported case of TaTME-assisted restorative proctocolectomy for FAP. While the narrow adolescent pelvis poses technical challenges for conventional approaches, it may theoretically enhance transanal visualization during the procedure. At two-year follow-up, the patient demonstrates good functional outcomes with 5–6 bowel movements per 24 h and maintained daytime continence.

**Conclusion:**

Laparoscopic restorative proctocolectomy utilizing TaTME technique appears feasible and safe in carefully selected adolescent FAP patients when performed by experienced teams. This approach may offer advantages for rectal dissection in the confined pediatric pelvis while maintaining excellent functional outcomes. Further studies are needed to validate these findings.

## Highlights

• To our knowledge, youngest reported use of TaTME technique in FAP patient based on systematic literature search
• High-grade dysplasia at age 13 necessitated immediate surgical intervention
• Narrow adolescent pelvis may enhance visualization during transanal approach
• Good functional outcomes achieved at 2-year follow-up
• Case demonstrates feasibility when performed by experienced teams with appropriate expertise

## 1 Introduction

Familial adenomatous polyposis (FAP) affects approximately 1 in 8000–10,000 individuals worldwide, representing one of the most challenging hereditary cancer syndromes [1]. This autosomal dominant condition, caused by germline mutations in the adenomatous polyposis coli (APC) gene, leads to the development of hundreds to thousands of colorectal adenomas beginning in adolescence. Without timely surgical intervention, the progression to colorectal cancer is virtually inevitable, typically manifesting by the fourth decade of life [1,2].

The optimal timing of prophylactic surgery in young FAP patients remains a subject of ongoing refinement. Current guidelines from the European Hereditary Tumour Group (EHTG-ESCP) and the National Comprehensive Cancer Network (NCCN) provide contemporary recommendations for FAP management [[2], [3], [4], [5]]. These guidelines emphasize individualized approaches to surgical timing, with European recommendations suggesting surveillance initiation at age 12 years, while American guidelines advocate for screening between ages 10–15. The decision for surgical intervention is tailored based on polyp burden, histological features, and genotype-phenotype correlations. This strategic window attempts to balance cancer prevention against quality of life implications during critical developmental years. However, for adolescents presenting with advanced disease features—particularly those under 16 years with high-grade dysplasia—the availability of less invasive surgical techniques may warrant reconsideration of established timing paradigms [6].

The surgical landscape for FAP has evolved dramatically over the past three decades. Traditional open surgery, while oncologically effective, imposed substantial morbidity on young patients through extensive abdominal incisions, prolonged recovery periods, and significant psychosocial burden during formative years [7]. The advent of laparoscopic techniques revolutionized pediatric colorectal surgery, with multiple series demonstrating reduced surgical trauma, superior cosmetic outcomes, and accelerated recovery [8]. These advances proved particularly valuable for adolescent patients navigating the complex challenges of chronic disease management alongside educational pursuits and social development.

The emergence of transanal minimally invasive surgery (TAMIS) and transanal total mesorectal excision (TaTME) has opened new frontiers in colorectal surgery [9]. Initially developed by Atallah and colleagues for local excision of rectal neoplasms, the transanal platform has evolved to enable complex resections through natural orifice access. Systematic reviews and international registry data from adult populations have validated the safety and efficacy of transanal approaches, demonstrating superior visualization of the distal rectum, reduced conversion rates, and excellent oncological outcomes when performed by experienced teams [10,11]. However, recent meta-analyses have also highlighted the importance of appropriate training and careful case selection [12].

Despite these technological advances, the application of transanal techniques in pediatric and adolescent populations has remained remarkably limited. Our comprehensive literature review reveals that the youngest reported patient undergoing TaTME-assisted proctocolectomy for FAP was 16 years old, as described in a German multicenter experience [13]. This age limitation likely reflects both the recent adoption of the technique and understandable caution regarding its application in younger patients. Nevertheless, accumulating experience with transanal approaches in other pediatric conditions suggests potential advantages that may be particularly relevant to the unique anatomical challenges of adolescent surgery [14,15].

This case report, prepared in accordance with the SCARE 2025 guidelines [16], describes the successful application of laparoscopic restorative proctocolectomy utilizing TaTME technique in a 13-year-old patient with FAP. Beyond establishing technical feasibility, this experience provides insights that may inform surgical approach selection in young patients facing this challenging diagnosis.

## 2 Case presentation

### 2.1 Patient history and clinical presentation

A previously healthy 13-year-old girl was referred to our surgical department from a regional hospital due to persistent gastrointestinal bleeding and severe anemia requiring intervention. She had presented to the referring hospital with a three-month history of intermittent hematochezia that had progressively worsened in both frequency and volume. Her parents initially attributed the bleeding to dietary factors, but became concerned when she developed progressive fatigue and exertional dyspnea that interfered with school attendance.

Physical examination revealed a well-developed adolescent (height 145 cm, weight 44 kg) with marked conjunctival pallor. Vital signs showed no tachycardia (heart rate 90 beats per minute) with normal blood pressure. Abdominal examination was unremarkable without palpable masses or hepatosplenomegaly. Digital rectal examination demonstrated bright red blood on the examining finger without palpable masses.

Laboratory evaluation revealed severe iron deficiency anemia with hemoglobin of 7.8 g/dL, mean corpuscular volume of 68 fL, ferritin of 8 ng/mL, and transferrin saturation of 6 %. Further history revealed that her mother had died of metastatic colorectal cancer at age 39, diagnosed only two years prior.

### 2.2 Endoscopic findings and clinical decision-making

**Fig. 1:**
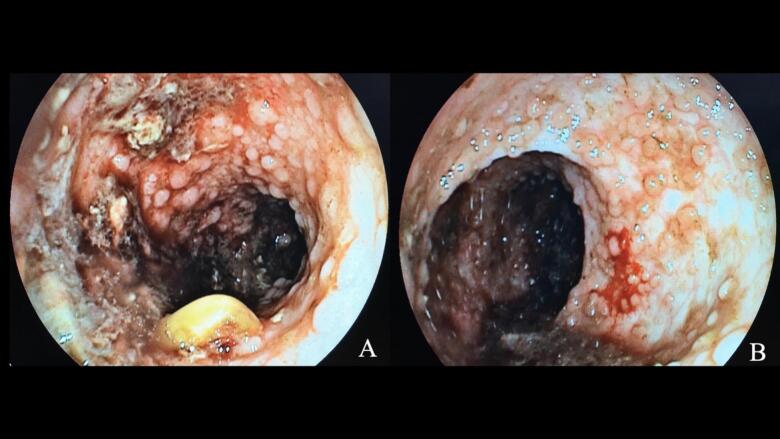
Preoperative endoscopic findings in 13-year-old FAP patient. (**A**) Sigmoid colon showing dense carpet-like polyposis with confluent adenomas and evidence of recent hemorrhage, demonstrating the characteristic left-sided predominance. (**B**) Rectum with extensive adenomatous polyposis, multiple polyps with villous architecture, and high-grade dysplasia confirmed on histopathology.

Urgent colonoscopy performed under general anesthesia revealed extensive adenomatous polyposis throughout the entire colon and rectum. Conservative estimates exceeded 500 individual polyps, ranging from 2 to 8 mm in diameter. The distribution showed marked heterogeneity—the cecum and ascending colon harbored numerous small sessile polyps (2–4 mm), while the sigmoid colon and rectum displayed confluent polyposis with larger lesions (Fig. 1A). This left-sided predominance pattern is characteristic of classical FAP. Several polyps exhibited spontaneous bleeding during the procedure, confirming the source of her chronic hemorrhage (Fig. 1B).

Targeted biopsies from 20 representative polyps revealed tubular adenomas with low-grade dysplasia in 15 specimens. Critically, high-grade dysplasia was identified in five separate locations—three within the rectum (at 3, 6, and 9 cm from the anal verge) and two in the sigmoid colon. No invasive carcinoma was detected. Upper endoscopy performed during the same session showed fundic gland polyps in the gastric body without duodenal involvement, classifying her as Spigelman stage 0 for upper tract disease [3].

Genetic counseling and testing confirmed a pathogenic frameshift mutation in the APC gene (c.3927_3931delAAAGA). This mutation, located within the mutation cluster region between codons 1250 and 1464, is associated with severe polyposis phenotypes and early cancer development [17,18].

Preoperative pelvic magnetic resonance imaging performed at the referring hospital demonstrated normal pelvic anatomy with an anteroposterior diameter of 8.2 cm, confirming anatomical suitability for pouch construction. No pelvic masses or congenital abnormalities were identified. Anorectal physiology studies demonstrated age-appropriate sphincter function, supporting the feasibility of continence preservation.

The convergence of multiple high-risk factors mandated immediate surgical intervention: symptomatic bleeding causing transfusion-dependent anemia, extensive polyposis precluding endoscopic management, multiple foci of high-grade dysplasia, and a severe APC mutation associated with aggressive disease [19,20]. The extensive rectal involvement with over 100 polyps and three separate areas of high-grade dysplasia definitively excluded ileorectal anastomosis as an option, necessitating total proctocolectomy with ileal pouch-anal anastomosis [6].

### 2.3 Preoperative planning and multidisciplinary assessment

Our multidisciplinary team convened to develop a comprehensive surgical strategy. The team included colorectal surgeons, pediatric gastroenterologists, pediatric anesthesiologists, geneticists, and pediatric psychologists. After thorough discussion, the decision was made to proceed with laparoscopic total proctocolectomy with ileal pouch-anal anastomosis, utilizing TaTME technique for the rectal dissection.

The rationale for employing the combined laparoscopic-TaTME approach emerged from several considerations. First, the narrow adolescent pelvis, which typically complicates conventional laparoscopic dissection, theoretically might favor the TaTME technique by providing enhanced “bottom-up” visualization. Second, the surgical team's extensive experience with TaTME in adults provided the technical foundation for adapting the approach to adolescent anatomy. Third, growing evidence suggests potential advantages of transanal approaches in confined pelvic spaces [21]. Comprehensive counseling addressed the novel nature of this approach in pediatric patients while emphasizing potential benefits including reduced abdominal trauma, superior cosmetic outcomes, and precise sphincter preservation. Written informed consent was obtained from both parents, and verbal assent was obtained from the patient.

### 2.4 Surgical technique

The procedure commenced with the patient in modified lithotomy position under general anesthesia. We employed a synchronized two-team approach, with simultaneous laparoscopic abdominal mobilization and transanal preparation, maximizing efficiency while minimizing operative time. Port placement was carefully planned to accommodate both the laparoscopic procedure and subsequent ileostomy creation. The total operative time was 280 min, with an estimated blood loss of 50 mL.

#### 2.4.1 Abdominal phase

**Fig. 2:**
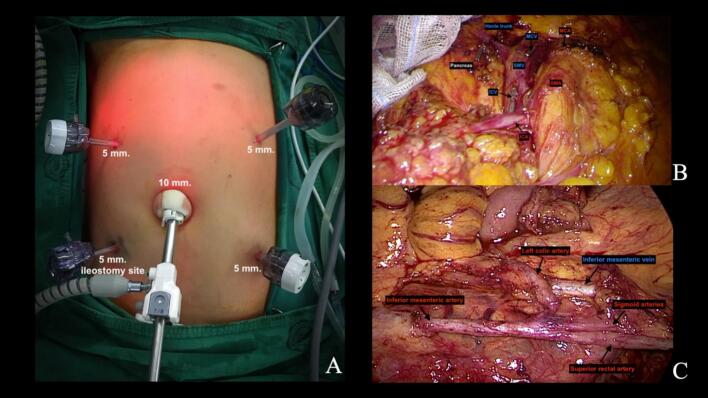
Laparoscopic abdominal mobilization phase. (**A**) Five-port laparoscopic setup showing 10-mm umbilical camera port, 5-mm ports in right upper quadrant, left upper quadrant, left lower quadrant, and right lower quadrant (planned ileostomy site). (**B**) Surgical field after completion of right and transverse colon mobilization showing the divided superior mesenteric vessel branches. (**C**) Inferior mesenteric vessels after dissection but prior to division, demonstrating the medial-to-lateral approach.

Five abdominal trocars were strategically placed to accommodate the smaller abdominal cavity—a 10-mm umbilical port for the camera, a 5-mm right upper quadrant port, a 5-mm left upper quadrant port, a 5-mm left lower quadrant port, and a 5-mm right lower quadrant port in the right iliac fossa (strategically positioned for later conversion to loop ileostomy) (Fig. 2A). Insufflation pressure was maintained at 10–12 mmHg to accommodate adolescent cardiovascular physiology.

The colonic mobilization employed a medial-to-lateral approach in two phases. First, we performed vascular control for the right and transverse colon by identifying and dividing the ileocolic, right colic, and middle colic vessels at their origins from the superior mesenteric vessels using an advanced dissecting device. Following this vascular control, we completed the lateral mobilization of the right colon, hepatic flexure, and transverse colon (Fig. 2B shows the surgical field after completion of right and transverse colon mobilization). The second phase addressed the left colon using the same medial-to-lateral principle. The inferior mesenteric artery and vein were identified and dissected at their origins (Fig. 2C). The splenic flexure was completely mobilized as part of the total colectomy. Throughout the dissection, we noted that the more delicate adolescent tissue planes required gentler handling compared to adult patients.

#### 2.4.2 Transanal phase

**Fig. 3:**
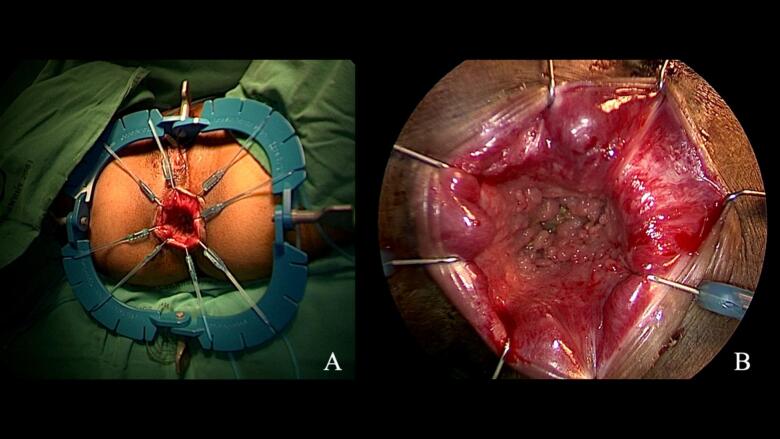
Transanal surgical setup and initial dissection. (**A**) Lone Star retractor system with smaller hooks utilized for anal exposure. (**B**) Zoom in picture shows multiple polyps at rectum.

**Fig. 4:**
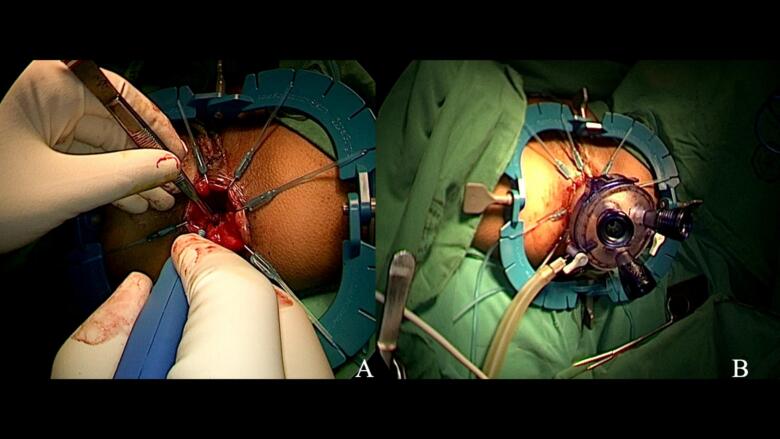
Transanal dissection technique. (**A**) Initial mucosectomy beginning 0.5 cm proximal to the dentate line, demonstrating precise dissection in the submucosal plane with excellent transanal visualization. (**B**) Transanal port placement in modified lithotomy position with specialized pediatric-sized instrumentation for optimal visualization of the anal canal and distal rectum.

Following completion of abdominal mobilization, we transitioned to the perineal portion. A Lone Star retractor with appropriately sized hooks provided circumferential exposure while avoiding excessive anal stretching (Fig. 3A,B). We performed circumferential purse-string closure of the distal bowel before initiating transanal dissection to prevent fecal contamination and maintain stable pneumorectum. The dentate line was carefully identified and marked circumferentially. Given the critical importance of sphincter preservation in this young patient, we initiated the mucosectomy just 5 mm proximal to the dentate line—closer than the typical 1–2 cm margin used in adults [9] (Fig. 4A).

**Fig. 5:**
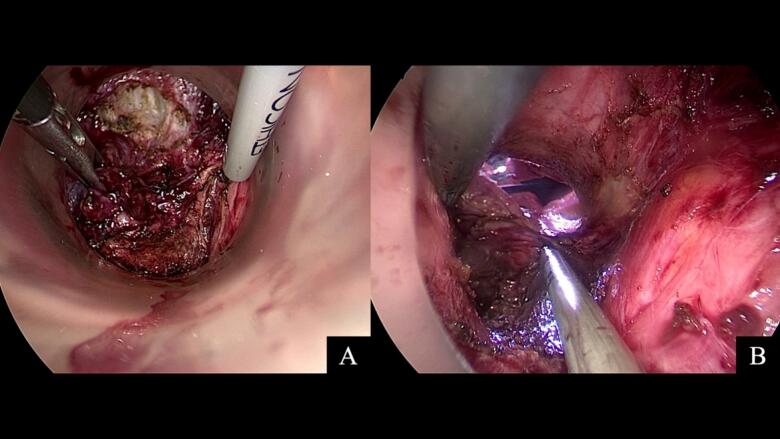
Transanal mesorectal excision technique. (**A**) Transanal visualization demonstrating superior access to the distal rectum with clear identification of tissue planes between mesorectum and pelvic fascia. (**B**) Progressive mesorectal dissection showing the precision achievable through direct transanal approach, with careful preservation of surrounding pelvic structures.

After establishing the initial mucosal cuff through careful submucosal dissection, we inserted a GelPOINT Path transanal access platform(Fig. 4B). The smaller adolescent anal canal required gradual dilation and careful positioning to avoid sphincter trauma. Once secured, we established pneumorectum initially at 8–10 mmHg with low flow (5 L/min) to minimize rectal spasm, gradually increasing to 12 mmHg as dissection progressed. A 5-mm 0-degree laparoscope provided optimal visualization. The circumferential rectal dissection proceeded in the “holy plane” - the avascular alveolar tissue plane between the mesorectal and presacral fascia (Fig. 5A). Following standard TaTME principles, we initiated dissection posteriorly at the 5–6 o'clock position where plane identification is most reliable, then proceeded laterally and finally anteriorly. The pelvic autonomic nerves, including the inferior hypogastric plexus and pelvic splanchnic nerves, were meticulously preserved bilaterally under direct visualization.

**Fig. 6:**
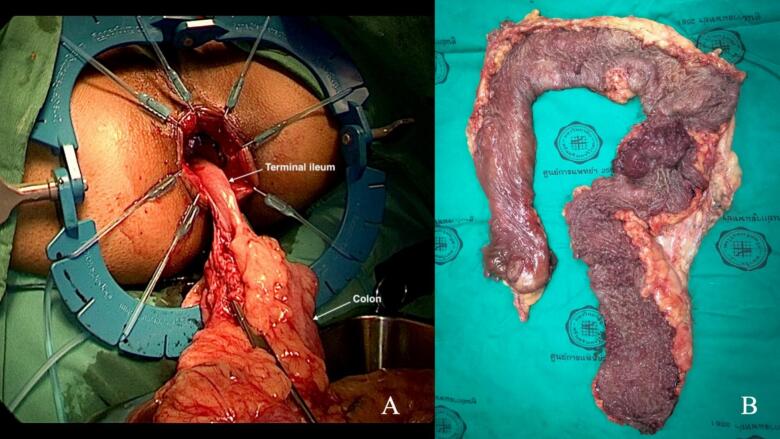
Specimen extraction and assessment. (**A**) Natural orifice specimen extraction through the anus. (**B**) Opened gross specimen demonstrating extensive polyposis throughout the entire colorectum with notably higher polyp density in the left-sided colon and rectum compared to the right colon, characteristic of the left-sided predominance pattern in classical FAP.

The anterior dissection in this female patient required exceptional precision to maintain the proper plane between the rectum and posterior vaginal wall. Using the transanal view, we clearly identified the rectovaginal septum. The dissection proceeded in the loose areolar tissue plane, avoiding both vaginal injury and violation of the mesorectal envelope. The “rendezvous” point where abdominal and transanal dissections meet was achieved at the peritoneal reflection (Fig. 5B). The specimen was delivered intact through the anal canal, eliminating the need for an abdominal extraction site (Fig. 6A).

#### 2.4.3 Reconstruction

Following specimen extraction, we created a 15-cm J-pouch using the terminal ileum. The pouch was constructed through the extended umbilical incision using a linear stapler. The pouch-anal anastomosis was performed using interrupted 3–0 polyglactin sutures under direct transanal visualization, ensuring precise approximation to the anal transition zone.

A diverting loop ileostomy was created based on several factors: (1) institutional protocol for all pediatric IPAA patients under 18 years, (2) technical complexity related to the narrow pelvic anatomy, (3) severe preoperative anemia potentially affecting tissue healing, and (4) extensive disease burden with multiple areas of high-grade dysplasia. While recent pediatric meta-analyses demonstrate that undiverted IPAA can be performed safely in selected cases [22], we opted for a cautious approach given these multiple risk factors. The stoma was fashioned through the marked right iliac fossa port site.

### 2.5 Postoperative course and pathological findings

The patient's recovery progressed smoothly. She experienced minimal postoperative pain, requiring only acetaminophen after the first 24 h. Clear liquids were initiated on postoperative day 1, with the ileostomy beginning to function on day 2. By postoperative day 3, she had advanced to a regular diet and was ambulating independently. Importantly, there was no further gastrointestinal bleeding, and her hemoglobin remained stable at 8.0–8.5 g/dL without transfusion. She was discharged on postoperative day 7 with comprehensive stoma education for both the patient and her parents.

Histopathological examination of the resected specimen confirmed extensive adenomatous polyposis with over 500 individual polyps identified by systematic counting. The distribution showed clear left-sided predominance, with the sigmoid colon and rectum containing approximately 70 % of the total polyp burden (Fig. 6B). High-grade dysplasia was confirmed in five separate polyps as previously identified on biopsy. No invasive carcinoma was detected. All surgical margins were negative for dysplasia. Eighteen examined lymph nodes showed only reactive changes without metastatic disease.

### 2.6 Stoma reversal and early outcomes

Ileostomy closure was performed at 10 weeks following the initial surgery. This timing, while earlier than traditional protocols suggesting 12–16 weeks, was based on careful assessment and emerging evidence. Pre-closure evaluation included water-soluble contrast enema demonstrating intact pouch-anal anastomosis without leak, stricture, or fistula. Clinical assessment with examination under anesthesia confirmed a healthy, well-vascularized pouch with good capacity.

This approach aligns with recent evidence from the Cleveland Clinic demonstrating that early reversal at 8–10 weeks is safe when performed as a planned, elective procedure. Their analysis of 2000 IPAA patients showed complication rates for planned early reversal (11.8 %) comparable to routine closure at traditional timing (11 %, *p* = 0.9) [23]. However, we acknowledge that early closure for stoma-related complications carries significantly higher risk, emphasizing the importance of careful patient selection.

The reversal procedure was completed in 50 min without complications. Initial functional results showed expected high stool frequency of 8–10 bowel movements daily, which gradually improved over the subsequent months. The patient was discharged on postoperative day 5 following demonstration of adequate oral intake and bowel function.

### 2.7 Long-term outcomes and follow-up

At two-year follow-up, the patient has achieved excellent functional outcomes. She reports 4–5 bowel movements during daytime hours with one nocturnal awakening, totaling 5–6 movements per 24-h period. Daytime continence is complete for both solid and liquid stool. Nighttime continence is good, with occasional nocturnal seepage (approximately once weekly) managed with a protective pad. She has returned to all school activities including competitive swimming, without functional limitations. Dietary tolerance is excellent without specific restrictions. Her hemoglobin has normalized at 12.5–13.0 g/dL without iron supplementation. These outcomes compare favorably with published pediatric IPAA series, as detailed in Table 1, which demonstrates that our results fall within or exceed expected ranges across all measured parameters including bowel frequency, continence rates, and complication rates.

**Table 1:** Comparative outcomes with pediatric IPAA literature.

Parameter	Our case	Literature range	References
Age at surgery	13 years	10–21 years	Shannon et al. [24]
Operative time	280 min	240–360 min	Kennedy et al. [20]
Hospital stay	7 days	7–10 days	Drews et al. [25]
Stoma reversal timing	10 weeks	12–16 weeks	Drews et al. [25]
**Functional outcomes at 2 years**			
Bowel frequency/24 h	5–6	4–8	Shannon et al. [24], Polites et al. [19]
Daytime continence	Complete^a^	80–95 %	Drews et al. [25], van Balkom et al. [7]
Nighttime continence	Good^b^	65–85 %	Drews et al. [25], Polites et al. [19]
**Complications**			
Early (<30 days)	None	15–25 %	Drews et al. [25]
Major (Clavien-Dindo ≥III)	None	3–7 %	Drews et al. [25]
Pouch failure at 2 years	No	3–8 %	Shannon et al. [24], Polites et al. [19]

a Complete continence for solid and liquid stool.

b Occasional nocturnal seepage managed with protective pad.

Quality of life assessment using the Cleveland Global Quality of Life score showed a result of 0.85 (where 1.0 represents perfect quality of life), comparing favorably with age-matched healthy controls. The patient reports high satisfaction with both functional outcomes and cosmetic results, with minimal visible scarring due to the laparoscopic approach and natural orifice specimen extraction.

Comprehensive surveillance has been established following international FAP guidelines [[3], [4], [5]]. Annual pouchoscopy shows healthy pouch mucosa without adenoma recurrence. The ileal mucosa demonstrates expected adaptive changes without dysplasia. Upper endoscopy surveillance commenced at age 14, revealing stable fundic gland polyps without progression. Additional screening includes annual thyroid ultrasound beginning at age 15 and clinical examination for desmoid tumors, with heightened vigilance given her young age at surgery and female gender.

## 3 Discussion

This case represents, to our knowledge based on systematic literature search through December 2024, the youngest reported application of TaTME technique for restorative proctocolectomy in FAP. The youngest previously documented cases were 16-year-olds described by Ambe and colleagues in their initial experience with TaTME for FAP [13]. While this three-year extension of the age boundary demonstrates technical feasibility, it also raises important considerations regarding patient selection, technical modifications, and long-term outcomes that merit detailed discussion.

### 3.1 Evolution of minimally invasive approaches in adolescent FAP

The surgical management of FAP in young patients has undergone remarkable transformation over the past two decades. Vitellaro and colleagues demonstrated the safety and efficacy of laparoscopic approaches in 38 adolescent patients with FAP, reporting only 5 % conversion rates and superior recovery compared to historical open controls [6]. These pioneering efforts established laparoscopy as the standard of care for pediatric colorectal resections, offering reduced postoperative pain, shorter hospital stays, improved cosmesis, and faster return to normal activities [8,23].

The landscape continues to evolve with emerging technologies. Single-incision laparoscopic surgery (SILS) has been successfully applied in adolescent FAP, offering potential cosmetic advantages through a hidden umbilical incision [21]. Robotic platforms provide enhanced three-dimensional visualization and improved dexterity, potentially advantageous in the confined spaces of pediatric anatomy [26,27]. However, despite these innovations, fundamental limitations persist—the “top-down” laparoscopic view struggles with visualization in the deep pelvis, a challenge particularly pronounced in the narrow confines of adolescent anatomy.

The transanal platform addresses these limitations through anatomical advantage. By approaching from below, surgeons gain unparalleled visualization of the critical distal dissection, precisely where conventional laparoscopy faces its greatest challenges [9,10]. International registry data from adult populations have validated this approach, with the first 720 cases demonstrating superior specimen quality, reduced conversion rates, and excellent functional outcomes [11,28]. However, the application of this technique to pediatric populations represents largely uncharted territory.

### 3.2 Technical advantages and anatomical considerations

The narrow adolescent pelvis, measuring 8.2 cm in anteroposterior diameter in our patient compared to typical adult measurements of 11–12 cm, initially appeared challenging. Paradoxically, this anatomical constraint may offer advantages during the transanal phase. The closer proximity of anatomical structures potentially enhances visualization, allowing more precise identification of tissue planes and critical structures [29]. Recent network meta-analyses comparing surgical approaches have demonstrated that TaTME offers superior visualization and reduced conversion rates specifically in patients with confined pelvic anatomy [30], supporting our technical choice for this challenging case.

Several technical modifications proved essential for success. Insufflation pressures were reduced to 12 mmHg to accommodate adolescent cardiovascular physiology. The mucosectomy commenced just 5 mm above the dentate line, closer than typical adult margins, to maximize sphincter preservation—a critical consideration for a patient with decades of functional requirements ahead. These subtle adaptations underscore the importance of surgical experience and judgment when applying adult techniques to younger patients.

### 3.3 Learning curve and expertise requirements

The learning curve for TaTME in adults is well-documented, requiring 40–51 cases to achieve proficiency, with complication rates inversely correlated with experience [31,32]. Structured proctorship can reduce this learning curve by approximately 30 %, but substantial experience remains essential [33]. This learning requirement raises important questions about the applicability of TaTME in pediatric populations, where case volumes are inherently lower.

Our team's extensive adult TaTME experience provided the technical foundation for this challenging case. However, the anatomical variations and technical modifications required for adolescent patients introduced new complexities. This suggests that TaTME in young patients should only be attempted by teams with substantial adult experience and in centers with appropriate multidisciplinary support. The concentration of expertise at specialized centers becomes even more critical when applying advanced techniques to rare pediatric cases.

### 3.4 Clinical implications and genotype-phenotype correlations

The exceptional presentation in our case—high-grade dysplasia at age 13—represents an aggressive phenotype documented in less than 1 % of FAP patients before age 16 [34]. The specific APC mutation (c.3927_3931delAAAGA) falls within the mutation cluster region associated with severe polyposis and early cancer development, validating the genotype-phenotype correlation [17,18]. This case exemplifies how genetic information can guide clinical decision-making in FAP management.

The distribution pattern observed—with predominant left-sided involvement and maximal severity in the rectosigmoid region—aligns with established patterns in classical FAP [35]. This distribution held particular surgical relevance, as the concentration of high-grade dysplasia in the rectum (three of five foci) definitively excluded ileorectal anastomosis as an option. While IRA generally offers superior functional outcomes, the presence of multiple high-grade dysplastic polyps in the rectum mandates total proctectomy according to current guidelines [3,6].

### 3.5 Functional outcomes in developmental context

The functional outcomes achieved—5-6 total bowel movements per 24 h with maintained continence—fall within the expected range for pediatric IPAA series. Comprehensive systematic reviews report bowel frequency ranging from 4 to 8 movements daily with daytime continence rates of 80–95 % [25]. Our patient's complete daytime continence and good nighttime control (with only occasional seepage) compare favorably with published outcomes. The absence of any early complications contrasts positively with reported rates of 15–25 % in pediatric series [25], though longer follow-up remains necessary to assess late complications.

These functional results gain particular significance when considering the patient's developmental trajectory. Unlike adults who may tolerate some functional compromise, adolescents face decades of educational, social, and professional activities where bowel function profoundly impacts quality of life [36]. Achievement of near-normal bowel habits enables comprehensive engagement in all aspects of teenage life—from sports participation to social activities to uninterrupted education. The psychological dimensions of surgery in adolescence warrant special consideration, with the superior cosmetic outcomes achievable through combined laparoscopic and transanal approaches addressing crucial body image concerns during these formative years [37].

### 3.6 Evidence base and current limitations

We acknowledge the limited high-level evidence for TaTME benefits specifically in pediatric populations. While the anatomical advantages appear logical, particularly in the narrow adolescent pelvis, randomized controlled trials comparing approaches in young patients are lacking. The theoretical benefits of improved visualization and nerve preservation require validation through prospective studies with long-term follow-up. Our case contributes to the emerging evidence base but cannot establish definitive superiority of the transanal approach.

Recent systematic reviews and meta-analyses of TaTME in adult populations have raised important considerations regarding oncological outcomes [12,38]. While these concerns primarily involve malignant disease and may not directly apply to benign conditions like FAP, they underscore the importance of appropriate training, careful patient selection, and meticulous surgical technique. The fundamental differences between adult rectal cancer and adolescent polyposis—including absence of mesorectal invasion, different tissue planes, and distinct healing characteristics—make direct extrapolation of oncological concerns less applicable to our population.

### 3.7 Future directions and broader implications

Current guidelines from both European and American societies continue to emphasize individualized surgical timing based on polyp burden and histological features [2,5]. Our case, with high-grade dysplasia at age 13, aligns with these guidelines' emphasis on prompt intervention when advanced pathology is present, regardless of age. The guidelines also stress the importance of specialized multidisciplinary care, which was integral to our approach.

As we consider broader applications of this technique, several factors warrant consideration. The success in this case establishes feasibility for transanal approaches in young adolescents with FAP, with potential applications extending to other conditions requiring proctectomy such as ulcerative colitis or juvenile polyposis syndrome [14,39]. However, careful patient selection remains paramount—candidates should have appropriate anatomy, disease characteristics warranting proctectomy, and access to experienced surgical teams.

The implications for surgical training and healthcare delivery are substantial. As minimally invasive platforms continue to evolve, the technical barriers to pediatric colorectal surgery may diminish. However, the substantial learning curve mandates careful consideration of centralization versus access, balancing expertise concentration against geographic barriers to care. This case exemplifies how specialized centers can advance surgical care while maintaining excellent outcomes.

### 3.8 Study limitations

Several limitations temper the generalizability of this single case report. The favorable outcome may reflect optimal circumstances—excellent general health, strong family support, and access to specialized multidisciplinary care. The two-year follow-up, while demonstrating good functional outcomes, cannot address long-term concerns regarding pouch durability, fertility impact, or cancer surveillance efficacy that require decades of observation. As a single case, we cannot determine complication rates or compare outcomes with conventional approaches. Future multicenter registries tracking pediatric transanal procedures would provide essential safety and efficacy data.

## 4 Conclusion

This case demonstrates the technical feasibility and safety of laparoscopic restorative proctocolectomy utilizing TaTME technique in a carefully selected 13-year-old patient with FAP. Based on our systematic literature review, this represents the youngest reported case of TaTME-assisted proctocolectomy for any indication. When clear clinical indications exist, as with our patient's multifocal high-grade dysplasia and symptomatic bleeding, technical innovation combined with appropriate expertise can enable optimal treatment without compromising functional outcomes.

The potential advantages of TaTME in the narrow adolescent pelvis, while theoretically appealing, require validation through larger studies with longer follow-up. Combined with meticulous technique and age-appropriate modifications, this hybrid approach achieved functional outcomes comparable to the best published pediatric series. As surgical technology advances and experience accumulates, such techniques may offer selected young FAP patients the best opportunity for cure while preserving quality of life.

However, this progress must be tempered with appropriate caution. Such procedures should only be performed at experienced centers with comprehensive multidisciplinary support and surgeons possessing substantial expertise in both minimally invasive colorectal surgery and adolescent care. This approach aligns with current international guidelines' emphasis on individualized, multidisciplinary management of FAP patients at specialized centers. As we expand the boundaries of surgical possibility, we must ensure that innovation serves our ultimate goal—enabling young patients with FAP to live full, productive lives free from the specter of colorectal cancer.

## CRediT authorship contribution statement

Hariruk Yodying: Conceptualization, data collection, writing - original draft, review and editing.

## Consent

Written informed consent was obtained from both parents and verbal assent from the patient for publication of this case report and accompanying images. A copy is available for review upon request.

## Ethical approval

This case report was conducted in accordance with the Declaration of Helsinki and reported following SCARE 2025 criteria. As per institutional policy, formal ethical approval was not required for this single case report.

## Guarantor

Hariruk Yodying accepts full responsibility for the work, had access to the data, and controlled the decision to publish.

## Research registration number

Not applicable - single case report.

## Funding

This research received no specific grant from funding agencies.

## Declaration of competing interest

The authors declare no competing financial interests.

## Data Availability

All data related to this case report are included in the manuscript.
